# Roughness Evolution and Charging in Plasma-Based Surface Engineering of Polymeric Substrates: The Effects of Ion Reflection and Secondary Electron Emission

**DOI:** 10.3390/mi9080415

**Published:** 2018-08-19

**Authors:** George Memos, Elefterios Lidorikis, George Kokkoris

**Affiliations:** 1 Institute of Nanoscience and Nanotechnology, National Center for Scientific Research “Demokritos”, Agia Paraskevi 15310, Greece; g.memos@inn.demokritos.gr; 2 Department of Materials Science and Engineering, University of Ioannina, Ioannina 45110, Greece; elidorik@cc.uoi.gr

**Keywords:** roughness, plasma etching, surface charging, ion reflection, secondary electron emission, simulation, modeling

## Abstract

The interaction of plasma with polymeric substrates generates both roughness and charging on the surface of the substrates. This work, toward the comprehension and, finally, the control of plasma-induced surface roughness, delves into the intertwined effects of surface charging, ion reflection, and secondary electron-electron emission (SEEE) on roughness evolution during plasma etching of polymeric substrates. For this purpose, a modeling framework consisting of a surface charging module, a surface etching model, and a profile evolution module is utilized. The case study is etching of a poly(methyl methacrylate) (PMMA) substrate by argon plasma. Starting from an initial surface profile with microscale roughness, the results show that the surface charging contributes to a faster elimination of the roughness compared to the case without charging, especially when ion reflection is taken into account. Ion reflection sustains roughness; without ion reflection, roughness is eliminated. Either with or without ion reflection, the effect of SEEE on the evolution of the *rms* roughness over etching time is marginal. The mutual interaction of the roughness and the charging potential is revealed through the correlation of the charging potential with a parameter combining *rms* roughness and *skewness* of the surface profile. A practical implication of the current study is that the elimination or the reduction of surface charging will result in greater surface roughness of polymeric, and generally dielectric, substrates.

## 1. Introduction

The investigation of plasma-induced surface roughness of polymers has emerged as a vital and substantial research area. Roughness not only affects, but also tunes, an amount of commercially valuable functional properties of the polymeric surfaces, such as optical properties and wettability [1,2,3] (e.g., fabrication of anti-reflective surfaces, superhydrophobic surfaces, self-cleaning surfaces, anti-bacterial surfaces). By the same token, the importance of roughness to stem cell differentiation [4,5,6] and, more generally, to cell-surface interactions [7,8,9,10], has also introduced plasma into the field of biomaterials and biomicrosystems. As a result, a series of former works [11,12,13,14,15,16,17,18,19,20] concentrated either on the mechanisms of roughness creation or on procedures eradicating or increasing roughness.

A phenomenon which is out of the discussion in the previous studies is surface charging. The latter is unavoidable during plasma etching of rough polymeric substrates as all requirements are there: the difference in the directionality between the positive ions and electrons bombarding the etched surface [21], the dielectric property of the substrates allowing charge to accumulate, and ultimately, the surface morphology, provoking the local inequality of positive and negative charges. There are measurements in previous studies verifying the existence of a surface charge density on the plasma etched polymeric substrates [22,23]. 

We have recently developed a modeling framework [24] for the evolution of rough polymeric surfaces under plasma consisting of a surface charging module [25], a surface etching model [26], and a profile evolution module [27]. It was applied to the investigation of the interaction between surface charging and microscale surface roughness of polymeric substrates [24]: tracking the evolution of an originally sinusoidal (rough) profile during plasma etching, it was revealed, on the one hand, that the surface charging assisted to the suppression of root mean square (*rms*) roughness and, on the other hand, that the lessening of the surface roughness caused a decrease of the charging potential. However, no mechanisms intensifying the pre-existing roughness were considered in that work [24]. In the current work, such a mechanism, namely ion reflection, is taken into account and integrated into the surface charging module of the modeling framework. Ion reflection is expected to enhance roughness [28] by increasing the flux of ions at the valleys of the surface morphology. In addition to ion reflection, the mechanism of secondary electron-electron emission (SEEE) is the second amendment to the surface charging module: an original model for the SEEE yield is developed for poly(methyl methacrylate) (PMMA) substrates in the energy range which is of interest in plasma etching. SEEE and the consequent electron redistribution on the dielectric surface could affect surface charging, as demonstrated in previous simulation studies on plasma etching of dielectric trenches [29,30]. The secondary ion-electron emission (SIEE) is not considered in the current work as it was found that, in the presence of SEEE, SIEE had an insignificant impact on the formation of the charging potential [30]. 

Toward the comprehension and, finally, the control of plasma induced surface roughness, the purpose of the current work, filling the relevant gap in the literature, is to record how charging is developed on the rough profile being etched and how it affects the evolving roughness of the profile, in the presence of ion reflection and SEEE. First efforts are also implemented in order to quantify the correlation between the surface roughness and the charging potential: the scaling of the charging potential to a combination of suitable statistical properties of the surface roughness is investigated. The objective of this work is attained by the modeling framework for the evolution of rough polymeric surfaces under plasma etching [24,25], which is properly extended to capture the effects of ion reflection and SEEE. The model system involves etching of a PMMA substrate by argon (Ar) plasma. The etching mechanism is physical sputtering by Ar^+^ ions.

The rest of this work is organized in the following way: in Section 2, the modeling framework is outlined. The model system and the case study are portrayed in Section 3. In Section 4, the interplay of surface charging, ion reflection, SEEE, and surface roughness is studied. The conclusions are summarized in Section 5.

## 2. The Modeling Framework

**Figure 1:**
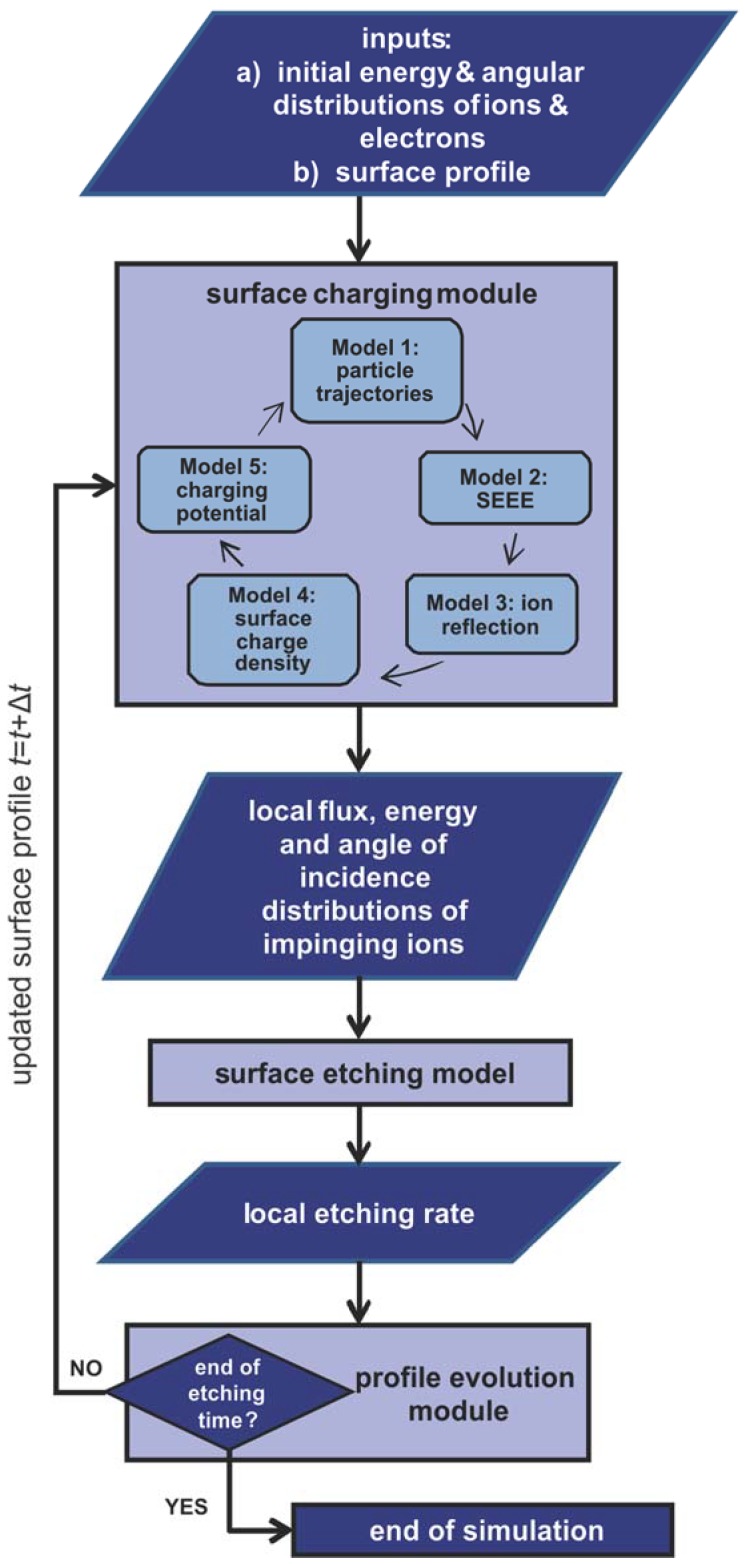
The modeling framework and the procedure of the computations. The coupling among modules of the framework, as well as the flow of data in the framework, is depicted.

The modeling framework is made up of (a) the surface charging module [25], (b) the surface etching model [26], and (c) the profile evolution module [27]. The coupling among modules of the framework is portrayed in the schematic diagram of Figure 1. The inputs are the energy and angular distributions of ions and electrons together with the initial surface profile. 

The simulation begins with the surface charging module where the local ion flux, as well as the distributions of ion energy and angle of ion incidence along the surface profile, are calculated. The outputs of the surface charging module are employed by the surface etching model to compute the local etching rate. The evolution of the surface profile is implemented by the feed of local etching rates to the profile evolution module. The surface profile is renewed, and the procedure is repeated until the total etching time is achieved. It has to be observed that the charging module is solved separately from the profile evolution module as the charging phenomenon progresses rapidly and reaches a steady state in a time much lower than the time step of surface profile evolution [24]. All models of the framework are coupled through a homemade code in Matlab (version 2018a) [31]. 

### 2.1. The Surface Charging Module

The surface charging module is utilized to treat the dynamics of charged particles exposed to a local electric field. The source of the field is a perpetually changing surface charge density. Charge is dropped on the surface during plasma etching due to the ion and electron impingement on the surface. The module (Figure 1) comprises of (a) a particle tracing model for the computation of ion and electron trajectories, (b) a SEEE model, (c) an ion reflection model, (d) a model for the computation of the local surface charge density, and (e) a model for the computation of the potential induced by the surface charge. A sequential run of the five models is redone until the charging potential attains a steady state. The assumptions of the surface charging module as well as three of its five models are described in depth in [25]. A short description of the particle trajectory, surface charge density, and charging potential models is included in Section 2.1.1, Section 2.1.4, and Section 2.1.5. The two new models, i.e., the SEEE model and the ion reflection model, are presented in depth in Section 2.1.2 and Section 2.1.3.

#### 2.1.1. Particle Trajectory Model

The particle trajectories are numerically calculated by solving a system of ordinary differential equations extracted from the second law of Newton and accompanied with the appropriate boundary conditions [25]. A multistep method with variable step size (namely ode15s of Matlab) is utilized. Parallel computing techniques, such as parallel loops and code vectorization, are used to scale down the computational time. A particle trajectory is terminated on the surface profile and the termination condition is implemented by utilizing the signed distance function from the surface profile. The signed distance function is calculated by the solution of the Eikonal equation [25] with the fast marching method [32]. The fast marching method is implemented with a homemade C++ code.

#### 2.1.2. Secondary Electron-Electron Emission Model

When an electron impinges on the PMMA surface, there are three potential events: it may stick to the surface, it may be reflected, or it may produce a secondary electron. This behavior can be described by the total electron yield *σ*_e_, equal to *δ* + *η*, which is commonly defined as the number of emitted electrons per incident (primary) electron. According to this definition, the yield includes three categories of emitted electrons [33]: (a) elastically reflected primary electrons, (b) inelastically reflected primary electrons, and (c) true secondary electrons. *δ* is the secondary electron emission yield including (c) and *η* is the backscattering coefficient including (a) and (b). All coefficients, *σ*_e_, *δ*, and *η*, may depend on the energy and the angle of incidence of the primary electrons, as well as on the substrate material. In the following, a model for *σ*_e_, *δ*, and *η* is described. It is based on available information in the literature for PMMA and other polymers in the energy range, which is of interest in plasma etching.

The initial electron energy distribution function (EEDF) is a Maxwellian distribution with electron temperature equal to 4 eV (cf. Section 3). The energy domain of such a distribution is extended approximately to 25 eV [25]. Nevertheless, the energy of an electron can be increased further due to acceleration by the developed charging potential. Given the electrostatic attraction, it is predicted that the range of energies of the primary electrons bombarding the PMMA surface will be from 0 to 50 eV. The same energy domain was also assumed during plasma etching of a SiO_2_ trench in view of SEEE [29,30].

**Figure 2:**
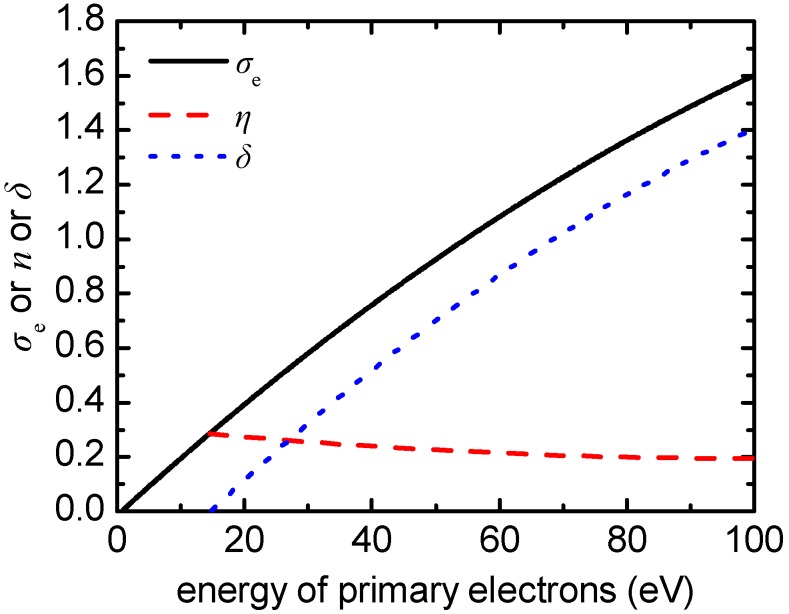
The total electron yield, *σ*_e_, the secondary electron emission yield, *δ*, and the backscattering coefficient, *η*, being utilized in the SEEE model.

Unfortunately, the literature is not deluged with publications describing *δ* and *η* for PMMA in the energy range from 0 to 50 eV. Few existing works concern mostly high energy electron bombardment of the PMMA surface. For instance, experimental data on *δ* for PMMA are available only for (primary electron) energies ranging from ~100 eV to several keV [34,35,36]. There is also one study including measurements of *η* in the energy range of 5 to 35 keV [33]. To the best of our knowledge, there are no experimental data that accurately portray the contribution of *η* to *σ*_e_ at low energies for PMMA. However, there are analytical expressions describing *δ* and *η* for the whole energy spectrum in the case of PMMA such as the Lin and Yoy law [37] for *δ*. Yu et al. [38] also proposed an analytical expression to describe *δ* and used an analytical equation derived by Burke [39] for *η*. Regarding the computational studies, Dapor et al. [40] developed a Monte Carlo model for the emission of secondary electrons from PMMA. They calculated *δ* in the energy domain ranging from a few keV down to a few tens of eV [40]. Dapor [41] also calculated the total electron yield *σ*_e_ as a function of the primary electron kinetic energy varying from 0 to 1500 eV. *σ*_e_ from the latter work is adopted in this work (Figure 2), as it is the only describing *σ*_e_ in the energy range of interest (0–50 eV).

In order to separate the backscattering proportion of electrons, we use the Burke’s equation [39] for *η*:

(1) $$\eta =0.115{\left(\frac{E_{0}}{10^{3}}\right)}^{-0.223}$$

where *E*_0_ is the energy of the primary electrons. Equation (1) was also utilized by Yu et al. [38] for PMMA. Generally, it expresses *η* in polymers consisting of H, C, N, and O as a function of *E*_0_ (eV). It should be mentioned that, in the energy range of interest (0–50 eV), we assume that *η* represents only elastically-reflected electrons. This simplification is prompted by Monte Carlo calculations for Teflon demonstrating that only elastically reflected electrons contribute to *η* for energy lower than 50 eV [42]. 

*η* from Equation (1) increases rapidly as the energy of the primary electron goes to zero and, strictly speaking, Equation (1) results in *η* > *σ*_e_ for 0 to 16 eV, something that is not realistic. A compromise is to consider that *n* below 16 eV is equal to *σ*_e_. Thus, *δ*, i.e., *σ*_e_ − *η*, is considered equal to zero for energy lower than 16 eV (Figure 2). It should be noted that the value of 16 eV is not far from the value of 12.6 eV, i.e., the average energy required to produce one secondary electron for PMMA [36]. It is also not far from the value of 10 eV, the general threshold for the secondary electron emission process [43]. For energy greater than 16 eV, *δ* is calculated as the difference of *σ*_e_ and *η* (Figure 2).

Although *δ* generally depends on the angle of electron incidence [40], this dependence is diminished in the energy range of interest (0–50 eV), as shown by both experimental [44] and simulation data [40]. Thus, it is considered that *δ* does not depend on the angle of electron incidence for energy range which is relevant for plasma etching.

Regarding the energy distribution of the secondary electrons, a typical secondary electron energy spectrum was presented by Dekker [43]. Nobuo et al. [45] calculated that the average energy of secondary electrons from PMMA was 15 eV when the energy of the primary electrons was 5 keV. Seiler et al. stated [44] that the energy distribution of secondary electrons, released by primary electrons with energies more than 100 eV is essentially independent of the primary energy and proposed an energy distribution of secondary electrons typical for insulating materials. The latter distribution is used by Seggern [46] for secondary electrons from Teflon and by Yu et al. [38] for secondary electrons from PMMA. Given the absence of data for the energy distribution of secondary electrons in the energy range of interest (0–50 eV), the energy of the secondary electrons is considered independent of the energy of the primary electrons and equal to the most probable energy of the distribution proposed by Seiler et al. [44,46] The energy of the secondary electrons is considered equal to 1 eV.

Regarding the angular distribution of secondary electrons, an isotropic (cosine) distribution is considered, following Monte Carlo calculations for PMMA [40] as well as experimental measurements for polycrystalline surfaces [44]. Finally, regarding the energy and the direction of the (elastically) reflected electrons, specular reflection with no energy losses is considered. The same approach is adopted for ions (see Section 2.1.3).

#### 2.1.3. Ion Reflection Model

Models for the reflection of ions on surfaces have been proposed [47,48,49,50,51,52,53] in the context of plasma etching of conventional structures of microelectronics (trenches and holes) to study etching artifacts due to surface charging, such as notching [47], twisting [53], and microtrenching [48]. Hwang and Giapis [47,48] assumed inelastic and specular reflection model for Si and silicon-on-insulator (SOI) substrates, following hard sphere collision kinematics. Specular and elastic reflection was considered by Zhao et al. [49] for photoresist and SiO_2_ substrates. Radmilovic-Radjenovic et al. [50] also considered specular reflection for SiO_2_ substrates. Wang and Kushner [53] considered both specular (at high energies) and diffusive (at low energies) reflection for SiO_2_ substrates. In all of the previous works [47,48,49,50,51,52,53] it was considered that the incident ions deposited their charge and were reflected as hot neutral species.

Following the previous works, and in the absence of experimental information on the detailed nature of the reflection of Ar^+^ on a PMMA surface, specular and elastic reflection of Ar^+^ is considered, although Ar^+^ may be implanted or may lose energy at the collision. Additionally, it is considered that ions drop their charge at the spot of the impact and are reflected as hot neutral species [47,53].

If **n** is the unit normal vector on the surface and **d** is the unit vector on the direction of the incident ion, the direction of a specularly-reflected ion is given by vector **r**, i.e.,:

**r** = **d** − 2 (**d·n**) **n** (2)

The probability of specular reflection is considered [26]:

*P* = 1 − cos*θ* (3)

where *θ* is the angle of ion incidence with respect to the normal to the surface.

#### 2.1.4. Surface Charge Density Model

For the calculation of the surface charge density, *σ*, the surface profile is divided into equal segments, and *σ* is calculated as the ratio of the local accumulated net charge (charge of impinging ions—charge of impinging electrons) over the segment length. *σ* links the particle trajectories and the electric field. The electric field not only regulates particle trajectories, but is also regulated by them via *σ*.

#### 2.1.5. Charging Potential Model

The space electric field and potential is calculated by the solution of the Laplace equation. Appropriate boundary conditions, such as the surface charge density (see Section 2.1.4), are applied [25]. The finite element method is utilized for the numerical solution; linear basis functions and a mesh of triangular elements are utilized. The numerical solution is implemented with COMSOL (version 5.0) [54]. 

### 2.2. Surface Etching Model

**Figure 3:**
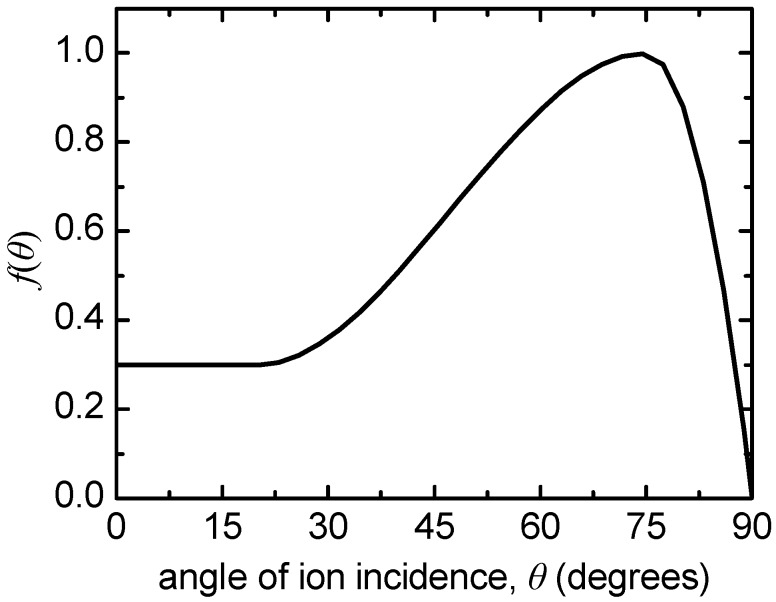
The function *f*(*θ*) of Equation (4) vs. the angle of ion incidence, *θ*. The maximum is at 75°.

The surface etching model is imposed locally on the evolving profile. In view of the fact that the model system in this work involves Ar^+^ ions impinging on a PMMA surface, the etching yield (the etching rate is the product of the ion flux with the etching yield) is expressed by [26]:

(4) $$EY=Af(\theta )\left(\sqrt{E_{+}}-\sqrt{E_{th}}\right)$$

where *E*_+_ is the ion energy and *E_th_* is the threshold ion energy for PMMA sputtering. *E_th_* is regarded equal to 4 eV and *A* equal to 0.1 monomers/(ion·eV^0.5^) [26]. *θ* in Equation (4) is the angle of incident ions. The angle dependence being manifested by *f*(*θ*) is depicted in Figure 3. It is typical for cases of physical sputtering [55,56] and is approximated by a simple polynomial function, the form of which can be found in [26].

### 2.3. Profile Evolution Module

The local etching rate calculated by the surface etching model is supplied to the profile evolution module which computes the successive positions of the profile. The profile evolution module is relied on the level set method [57,58]. Details for the module are included in Reference [27]. The calculations are implemented by φetch code [59] after appropriate extensions treating surface charging and unconventional surface profiles.

## 3. Case Study

**Figure 4:**
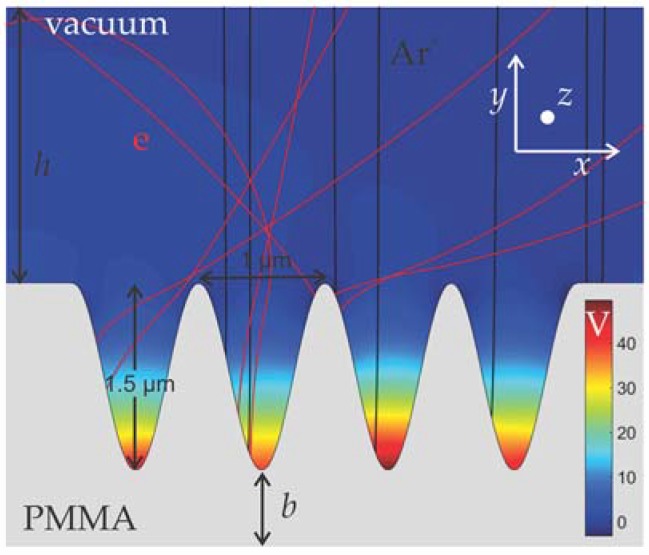
The initial sinusoidal profile of the PMMA surface. The steady state potential, as well as some of the ion (in black) and electron (in red) trajectories, are also depicted. *h* is the height of the vacuum space, and *b* is the thickness of the PMMA layer. *h* is 2.2 μm, and *b* >> *h*.

The case study is plasma etching of a PMMA substrate with a sinusoidal, simulating a rough, profile (Figure 4). The etching mechanism is physical sputtering by Ar^+^. The ion energy and angle distribution functions (IEADFs) for Ar^+^, as well as the electron energy and angle distribution functions (EEADFs) are the same as in [25,60]. The mean energy of ions and electrons are 90 and 4 eV, respectively. The ion angular distribution resembles a Gaussian and the electron angular distribution is isotropic. The ion flux is 1.86 × 10^20^ m^−2^·s^−1^. The dielectric constant of PMMA is equal to 3. A substrate with a high (infinite) thickness is considered. The role of the substrate thickness on charging has been analyzed previously [24] and it has been found that although the thickness of the dielectric substrate affected the charging time, i.e., the time required for reaching a steady state charging potential, it did not affect the roughness evolution: the charging time was much shorter than the etching time for all values of thickness (0.1 μm to infinite thickness for surface roughness at the microscale).

## 4. Results and Discussion

Three mechanisms are considered responsible for intertwining with the roughness evolution, i.e., ion reflection, surface charging, and SEEE. They are reviewed separately in terms of numerical models, which evaluate the corresponding importance of each mechanism on the total process of roughness evolution. The purpose of the simulation is to quantify the effect and, thus, to identify the role of the individual mechanisms on the roughness evolution.

**Figure 5:**
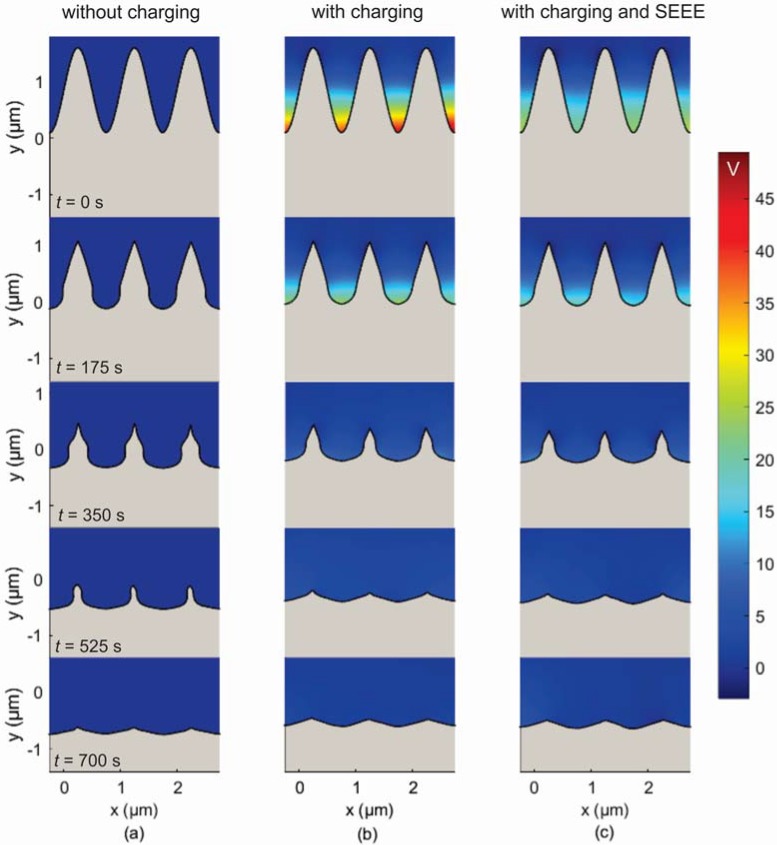
Snapshots of the surface profile for different etching times (**a**) without charging (multimedia view, please see Figure5a.avi), (**b**) with charging (multimedia view, please see Figure5b.avi), and (**c**) with charging and SEEE (multimedia view, please see Figure5c.avi), when the ion reflection is not taken into account. The profiles are cut from the middle of the first valley to the middle of the last one. The charging potential for the snapshots of Figure 5b,c is also depicted.

In Figure 5, the profile evolution of the initial sinusoidal profile in the absense of ion reflection is depicted. Charging is ommited in Figure 5a, while, it is considered in Figure 5b; the charging potential is also included in Figure 5b. It is shown that the profile peaks are almost eliminated at the final stage (*t* > 525 s) either when charging is considered or not. The latter is attributed [24] to the strong angle dependence of the etching rate which mitigates the effects of charging. Although the etching (sputtering) yield depends on both the ion energy and the angle of ion incidence (Equation (4), Figure 3), the effect of ion incidence, and as consequence of the profile slope, dominates. This dominance originates from the big increase of the etching yield at angles of ion incidence in the range of 60°–80°. In case there is no angle dependence, a different behavior is expected [24].

It has to be noticed that the peaks are reduced slightly faster in the case of charging (cf. Figure 5a,b). The joint action of the ion deflection toward the sidewalls with the attenuation of the ion energy at parts of lower *y* coordinate induces a smaller slope to the sidewalls compared to the case without charging (Figure 5a). Consequently, the angle of ion incidence at the sidewalls of the peaks is smaller in the case of charging approaching the maximum of the etching yield (Figure 3). Thus, the local etching rate is higher at the sidewalls, and subsequently, the profile peaks are eradicated at a faster pace when charging is involved in the process.

In Figure 5c, besides charging, SEEE is also included. Comparing it with Figure 5b, hardly any differences are distinguished in the evolving profiles. However, the charging potentials at *t* = 0 s differ. The initial profile (Figure 5b, *t* = 0 s) induces heavy geometric shadowing in the isotropic electron flux. Most electrons impinge on the upper region of the surface sidewalls. As the positive potential is developing in the valley region, electrons are attracted there in order to compensate the overwhelming initial current imbalance. In the absence of SEEE, such current balance is attainable for a potential of 45 V. With the inclusion of SEEE (Figure 5c, *t* = 0 s) the charging potential is reduced ~50% because a larger electron flux impinges at the valley region. Indeed, due to the emerging positive potential, it is more probable for a secondary (or a reflected) electron to terminate its trajectory at the valley region during the charging process. Thus, in order for current balance to be restored, a lower potential is needed. However, the effect of the SEEE in the charging development is mitigated as the profile evolves and eventually it is disappeared for *t* > 175 s (cf. Figure 5b,c at 175 s and 350 s). After this time, the profile valleys are wide enough to reduce the electron shadowing and receive the great majority of the incident electrons. Hence, the charging potential is the same to that in the absence of SEEE (cf. Figure 5b,c at 350 s).

**Figure 6:**
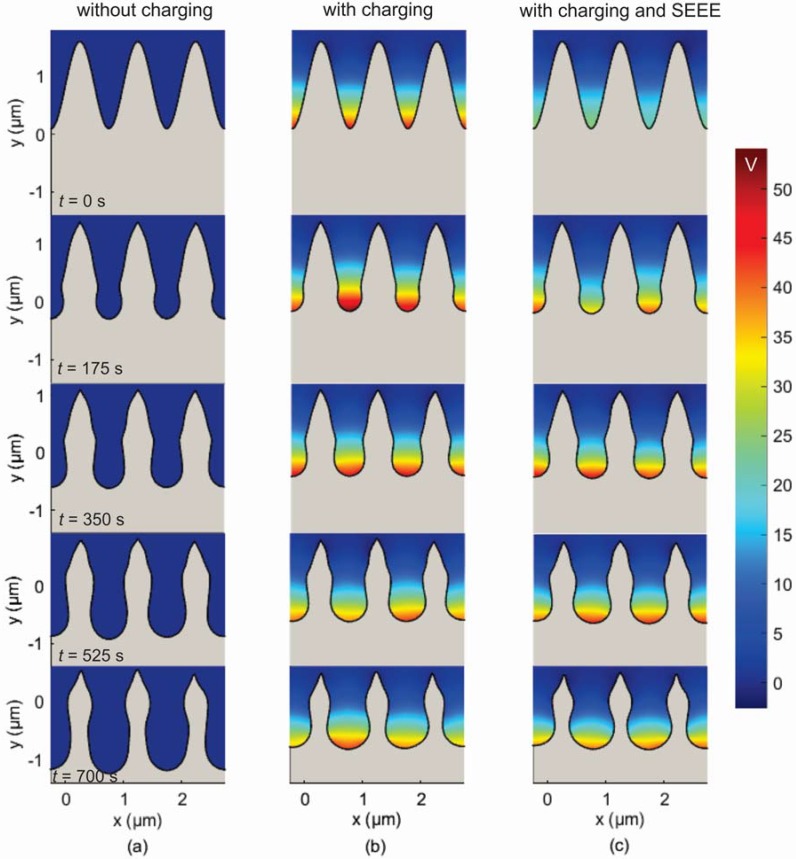
Snapshots of the surface profile for different etching times (**a**) without charging (multimedia view, please see Figure6a.avi), (**b**) with charging (multimedia view, please see Figure6b.avi), and (**c**) with charging and SEEE (multimedia view, please see Figure6c.avi), when the ion reflection is taken into account. The profiles are cut from the middle of the first valley to the middle of the last one. The charging potential for snapshots of Figure 6b,c is also depicted.

In Figure 6 the same results as in Figure 5 are shown, including the ion reflection mechanism. Charging is omitted in Figure 6a, while it is considered in Figure 6b and, ultimately, in Figure 6c, besides charging, SEEE is also taken into account. First, by comparing Figure 5 and Figure 6, it is shown that when the ion reflection is taken into account, the profile features are sustained until the end of the etching time, i.e., roughness is not eliminated. Second, when charging is taken into account, the peaks of the profile are shorter and thinner (cf. Figure 6a,b). Third, as in the case without ion reflection, although SEEE initially (*t* < 175 s) induces a decrease of the charging potential, there are no apparent differences in the profiles with and without SEEE (cf. Figure 6b,c). This is because the available surface for electron reflection or emission towards the valleys is reduced. The SEEE mechanism just redistributes the incident electrons locally in the profile valleys and this local redistribution does not significantly affect the electron flux (and, hence, the electric potential).

**Figure 7:**
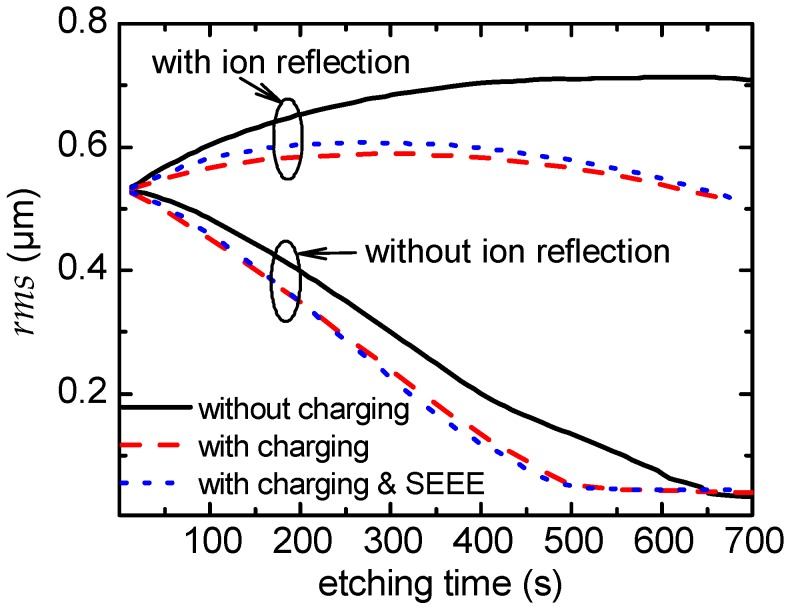
The *rms* roughness vs. the etching time for the surface profiles shown in Figure 5 and Figure 6.

The *rms* roughness of the evolving profiles in Figure 5 and Figure 6 versus the etching time is shown in Figure 7. The eradication of the profile peaks when ion reflection is not taken into account (Figure 5) is manifested by the decrease of the *rms* roughness versus time. The slightly faster eradication of the peaks due to charging is substantially quantified by the marginally faster attenuation of the *rms* roughness. When ion reflection is taken into account (Figure 6) and in the absence of charging, the *rms* roughness increases initially but finally comes to a saturation as the “competitive forces of the process”, i.e., the angle dependent physical sputtering and the ion reflection, come to a balance. When both charging and ion reflection are considered, the *rms* roughness initially increases and after approximately 250 s starts to fall. Charging not only restrains the *rms* roughness at the initial stages of etching but, subsequently, it induces a decrease of the *rms* roughness. Finally, as demonstrated in Figure 7, either with or with ion reflection, the effect of SEEE on the evolution of the *rms* roughness over etching time is marginal.

The correlation of the surface charging potential with the profile roughness has been demonstrated in previous works of ours [24,25]. We have shown that charging is controlled by the aspect ratio for a sinusoidal profile [25]. The aspect ratio is determined as the ratio of two times the amplitude over the half period of the profile and reflects the significance of the electron shadowing effect. However, the aspect ratio cannot be defined for random rough profiles emerging in plasma based surface engineering applications. In an attempt to quantify the significance of the electron shadowing effect of such profiles on the surface charging potential, parameter *m* is proposed, which reads:

(5) $$m=\left(\frac{rms}{\lambda }\right)\frac{1}{c+skewness}$$

where *λ* is the distance between the surface peaks. The *skewness* of the profile quantifies the asymmetry of the distribution of the profile heights with respect to the mean profile height [61]. Generally, a surface with bumps has a positive skewness while a surface with holes has a negative skewness [62,63]. *c* in Equation (5) is a unitless constant with a positive value (1/2 in this work) so as to avoid division by zero or very large values when the *skewness* approaches 0. *m* quantifies the competitive effect between the ratio *rms*/*λ* and the *skewness* on the electron shadowing. Electron shadowing is enhanced when the ratio *rms*/*λ* increases, i.e., when the surface features (e.g., bumps, peaks or holes) are greater and at close range. Electron shadowing is also expected to be heavier for lower *skewness*, i.e., for surface profile comprised mainly by valleys (or a surface morphology comprised mainly by holes).

**Figure 8:**
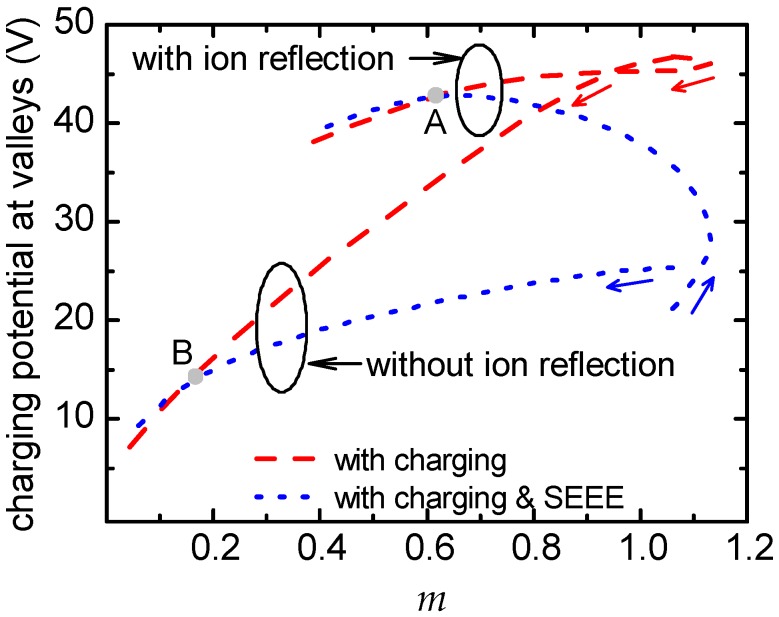
The charging potential (average value of potential at the four valleys of the profile) vs. parameter *m* (Equation (5)). The arrows on the curves denote the time path during etching. Values above 500 s of etching for the curves corresponding to cases without ion reflection have been removed as the profiles are almost flat (cf. Figure 7). The small difference (~3 V) observed in the initial potential between the two cases of SEEE is an expected difference between two runs of a stochastic process.

Figure 8 includes the charging potential at the bottom of the valleys (average value at the four valleys) versus *m* for the evolving profiles shown in Figure 5 and Figure 6. The arrows on the curves denote the time path during etching. Without ion reflection, the charging potential decreases with the decrease of *m*, either SEEE is taken into account or not. With ion reflection and without SEEE, there is an almost linear correlation between the potential and *m*. This linear correlation is disturbed when SEEE is taken into account and is restored when the SEEE effect fades away: the two curves after point A almost coincide. This coincidence is also true for the curves without ion reflection after point B. Figure 8 demonstrates that, besides the different mechanisms and phenomena taken into account (with or without SEEE, with or without ion reflection), the mutual interaction between surface charging and profile roughness is present in all cases examined.

## 5. Conclusions

Toward the comprehension and, finally, the control of plasma-induced surface roughness, we delved into the intertwined effects of ion reflection, surface charging, and SEEE on roughness evolution during physical sputtering of a PMMA substrate with argon plasma. For this, a modelling framework for profile evolution of polymeric surfaces under plasma etching was utilized. The framework was extended to include SEEE and ion reflection.

Regarding the SEEE, a model for the secondary electron emission yield, *δ*, and the backscattering coefficient, *n*, was developed combining available information for PMMA and other polymers in the energy range which is of interest in plasma etching. Regarding the reflection of Ar^+^ ions on a PMMA surface, a simple model of non-interacting collisions was considered, i.e., specular reflection of the ions on the surface with no energy losses.

Starting from an initial surface profile with microscale roughness, the calculations showed that the surface charging contributed to a faster roughness elimination compared to the case without charging. For the cases studied, the effect of SEEE on the evolution of *rms* roughness was marginal. When the ion reflection was considered, the profile features were preserved until the end of the etching time, i.e., roughness was not eliminated. In that case, charging not only constrained the *rms* roughness at the initial stages of etching but, afterwards, it led to a decrease of the *rms* roughness. 

The charging potential was correlated to the profile roughness through a parameter which suitably combines statistical properties of the profile, such as *rms* roughness and *skewness*. Regardless of the mechanisms and the phenomena taken into account, the charging potential showed an almost monotonic behavior with this parameter, something that revealed the mutual interaction between surface charging and profile roughness.

A practical implication of the current study is that the elimination or the reduction of surface charging will result into greater surface roughness of polymeric, and generally dielectric, substrates. The means to reduce surface charging is by increasing the flux of negative charges to the valleys of the surface profile. The latter can be achieved either by pulsed plasmas [64,65], which can produce and inject negative ions into the valleys, or by DC-augmented capacitive coupled plasmas [53,66], which can produce and inject narrow angle and high energy electrons into the valleys. An alternative to eliminate surface charging is by neutral beam etching [67,68,69].

From a computational point of view, the results of the current study suggest that the effect of surface charging should be taken into account in the design of recipes for producing or eliminating surface roughness. Finally, it has to be noticed that the plasma induced surface charging, not only affects the surface roughness developed on the etched surface, but it is also expected to affect the properties of the etched surfaces. The surface charge density developed on the etched surface was found to be stable [22] and affect the wetting properties of polymeric surfaces [23]. 

Although this work is the first attempt to link the surface charging with the surface roughness evolution of polymeric substrates in the presence of ion reflection and SEEE, the results of the simulations illustrate qualitative trends for the surface roughness. In order to produce quantitative results and to compare them with available experimental measurements, extension of the modeling framework will be required. Usually the measurements refer to surface roughness statistics (e.g., *rms*) at different operating conditions (e.g., pressure, flow rate, power). Thus, the comparison of the simulation results with measurements requires the extension of the modeling framework with a reactor scale model, i.e., it requires a multiscale modeling framework linking the operating parameters with the surface roughness [26]. The extension may also include additional mechanisms, such as product re-deposition [26], which can affect roughness evolution, or more detailed models, e.g., for the interaction of ions with the polymeric substrates. Finally, the modeling framework will be applied to plasma etching of polymeric substrates with O_2_ chemistry, which is widely used for the surface modification and roughening of polymeric substrates.

## Supplementary Materials

The following are available online at http://www.mdpi.com/2072-666X/9/8/415/s1, Video S1: Figure5a.avi, Video S2: Figure5b.avi, Video S3: Figure5c.avi, Video S4: Figure6a.avi, Video S5: Figure6b.avi, Video S6: Figure6c.avi.
